# Post-traumatic stress symptoms in intensive care staff working in adult and paediatric settings

**DOI:** 10.1186/cc14611

**Published:** 2015-03-16

**Authors:** G Colville, J Hammond, L Perkins-Porras

**Affiliations:** 1 St George's Hospital, London, UK; 2 St George's University of London, UK

## Introduction

The objectives of this survey were to establish the prevalence of symptoms of post-traumatic stress in mixed staff groups working in adult and paediatric intensive care settings and to examine the main themes in staff descriptions of the most traumatic event they had experienced at work.

## Methods

A total of 355 health professionals working on three adult and four paediatric units at two centres were asked to rate their current level of post-traumatic stress symptoms on the Trauma Screening Questionnaire (TSQ).

## Results

**Figure 1:**
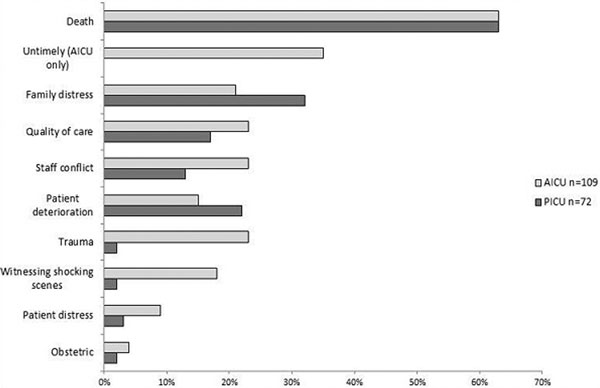
**Main themes of staff descriptions of traumatic events on ICU**.

Paediatric/neonatal intensive care staff were more likely to score above the clinical cutoff point for post-traumatic stress symptoms on the TSQ in relation to an incident at work than adult intensive care staff in this sample (PICU *n *= 33/193 (17%) vs. AICU *n *= 13/162 (8%), *P *< 0.001). For the 172 staff who provided a description of the most traumatic event they had experienced, the following themes were most commonly endorsed: patient death (and particularly untimely deaths on adult units); handling distressed families; and concerns about the quality of care and dealing with staff conflict (see Figure 1).

## Conclusion

A significant minority of staff reported clinically significant levels of post-traumatic stress related to their work. The facts that post-traumatic stress levels were higher on paediatric units despite their lower rates of mortality and that untimely deaths were frequently mentioned by adult unit staff suggest it may be that untimely deaths are particularly hard to deal with. More research is needed on the prevalence of distress in staff working in these settings.

